# *flyDIVaS*: A Comparative Genomics Resource for Drosophila Divergence and Selection

**DOI:** 10.1534/g3.116.031138

**Published:** 2016-05-25

**Authors:** Craig E. Stanley, Rob J. Kulathinal

**Affiliations:** Department of Biology, Temple University, Philadelphia, Pennsylvania 19122

**Keywords:** conserved genes, rapid evolution, *d_N_*/*d_S_*, adaptation

## Abstract

With arguably the best finished and expertly annotated genome assembly, *Drosophila melanogaster* is a formidable genetics model to study all aspects of biology. Nearly a decade ago, the 12 *Drosophila* genomes project expanded *D. melanogaster*’s breadth as a comparative model through the community-development of an unprecedented genus- and genome-wide comparative resource. However, since its inception, these datasets for evolutionary inference and biological discovery have become increasingly outdated, outmoded, and inaccessible. Here, we provide an updated and upgradable comparative genomics resource of *Drosophila* divergence and selection, *flyDIVaS*, based on the latest genomic assemblies, curated FlyBase annotations, and recent OrthoDB orthology calls. *flyDIVaS* is an online database containing *D. melanogaster*-centric orthologous gene sets, CDS and protein alignments, divergence statistics (% gaps, *d_N_*, *d_S_*, *d_N_*/*d_S_*), and codon-based tests of positive Darwinian selection. Out of 13,920 protein-coding *D. melanogaster* genes, ∼80% have one aligned ortholog in the closely related species, *D. simulans*, and ∼50% have 1–1 12-way alignments in the original 12 sequenced species that span over 80 million yr of divergence. Genes and their orthologs can be chosen from four different taxonomic datasets differing in phylogenetic depth and coverage density, and visualized via interactive alignments and phylogenetic trees. Users can also batch download entire comparative datasets. A functional survey finds conserved mitotic and neural genes, highly diverged immune and reproduction-related genes, more conspicuous signals of divergence across tissue-specific genes, and an enrichment of positive selection among highly diverged genes. *flyDIVaS* will be regularly updated and can be freely accessed at www.flydivas.info. We encourage researchers to regularly use this resource as a tool for biological inference and discovery, and in their classrooms to help train the next generation of biologists to creatively use such genomic big data resources in an integrative manner.

Rates of phenotypic divergence vary greatly between functional classes. In many cases, functional divergence reflects the evolutionary rates of their underlying genes and proteins (Castillo-Davis *et al.* 2004; Lemos *et al.* 2005; Janecka *et al.* 2012). For example, conserved cellular processes such as growth, metabolism, and replication are encoded by some of the slowest evolving genes, alignable across kingdoms (Zhang and Li 2004; Peregrín-Alvarez *et al.* 2009). At the other end of the divergence spectrum, rapidly evolving immune-related genes in animals underlie highly dynamic host-parasite interactions that often lack traceable orthologs (Clark and Lazzaro 2012). Similarly, fast evolving sex-related genes code for highly diverged traits involved in sexual dimorphism, reproductive isolation, and species differences and are common across sexual taxa (Wyckoff *et al.* 2002; Swanson and Vacquier 2002; Singh *et al.* 2012).

While the comparison of aligned sequences between species provides a complementary molecular approach to study organismal diversity, it also differentiates the two faces of selection—negative and positive—acting on biological processes. Patterns of nucleotide divergence tell us much about the fitness effects of mutational perturbations on proteins and their associated functional systems. Extending our current comparative framework to the level of the codon, both the strength and direction of selection can be inferred by comparing the ratio of nonsynonymous substitutions per nonsynonymous site (*d_N_*) to synonymous substitutions per synonymous site (*d_S_*) (Li *et al.* 1985; Saitou and Nei 1987). Genes harboring low *d_N_*/*d_S_* ratios reflect high levels of protein conservation across species as negative selection preserves protein function for conserved processes. Genes with orthologous codons exhibiting high *d_N_*/*d_S_* suggest that positive selection quickly drives the fixation of amino acids as organisms better adapt to their surroundings and to each other. Alternatively, high *d_N_*/*d_S_* may indicate a less substantive role of selection on preserving protein function.

The increasing availability of genome assemblies has now made this molecular evolutionary framework a cornerstone of comparative and functional genomic analysis. Coupled with ever-expanding functional annotations (*e.g.*, gene ontologies, tissue, developmental stage, etc.), we have increasing power to detect divergence signatures across biological processes. In fact, some of the most interesting findings from genome projects are the validation and/or discovery of new evolutionary patterns that illuminate the adaptive history of the sequenced species (*e.g.*, Stapley *et al.* 2010; Radwan and Babik 2012). Various substitution models of *d_N_*/*d_S_* evolution can also yield insight into the site heterogeneity of protein stasis and change, thus providing solutions to diverse biological problems ranging from conservation management (Crandall *et al.* 2000; Stockwell *et al.* 2003) to drug targeting and production (Allen *et al.* 2014). However, this comparative functional framework depends on a highly accurate set of assemblies with precise gene models.

Over the last 15 yr, *Drosophila* has transformed from a premiere genetic model into among the most powerful genomic models with unprecedented resources and tools for comparative (Clark *et al.* 2007), population (Mackay *et al.* 2012), and functional (Celniker *et al.* 2009; Robinson *et al.* 2013; dos Santos *et al.* 2015) genomics. *D. melanogaster* was among the first eukaryotes with a “finished” genome (Hoskins *et al.* 2015) and expertly curated gene models across the phylogeny (dos Santos *et al.* 2015). Over a decade ago, fruit fly researchers from diverse fields collaborated to assemble, align, and annotate a dozen species of *Drosophila* spanning 80 million yr of evolution (Clark *et al.* 2007). An online resource known as the *Drosophila* AAA (Assembly, Alignment, Annotation) site was developed and curated by the *Drosophila* community as a temporary measure to provide immediate community access to this unique comparative genomics resource. As of 2016, over 1200 papers have cited the original Clark *et al.* (2007) publication, and researchers continue to analyze results from this important dataset even though *D. melanogaster* has undergone two major genomic assembly revisions, numerous genomic releases in the other sequenced species, and more than 50 annotation updates.

Here, we present an updated, comprehensive comparative genomics resource of divergence and selection on protein-coding genes in 12 species of the genus *Drosophila*. *flyDIVaS* will be regularly updated in synchrony with the latest gene models from FlyBase and orthology calls from OrthoDB. Users will be able to choose between four taxonomic datasets (Figure 1) covering different phylogenetic and sequence depths. *flyDIVaS_v1* provides: 1) 1:1 orthologous gene sets, 2) CDS and protein alignments including gap-masked alignments, 3) *d_N_*/*d_S_* estimates, and 4) results from codon substitution (site-specific) selection tests. Alignments and their resulting phylogenetic trees can be visualized online through interactive graphical features. We also present a preliminary analysis of divergence and selection across functional ontological categories and confirm previous observations of high immune and reproductive gene divergence, with stronger signal in genes that are tissue-specific. While highly diverged proteins are enriched in positive selection in the testis and ovary, they appear to be neutrally evolving in accessory glands. Our primary objective is to provide both researchers and students a freely available, gold-standard platform to explore the divergence and adaptive landscape across nearly 100 million yr of evolution.

**Figure 1:**
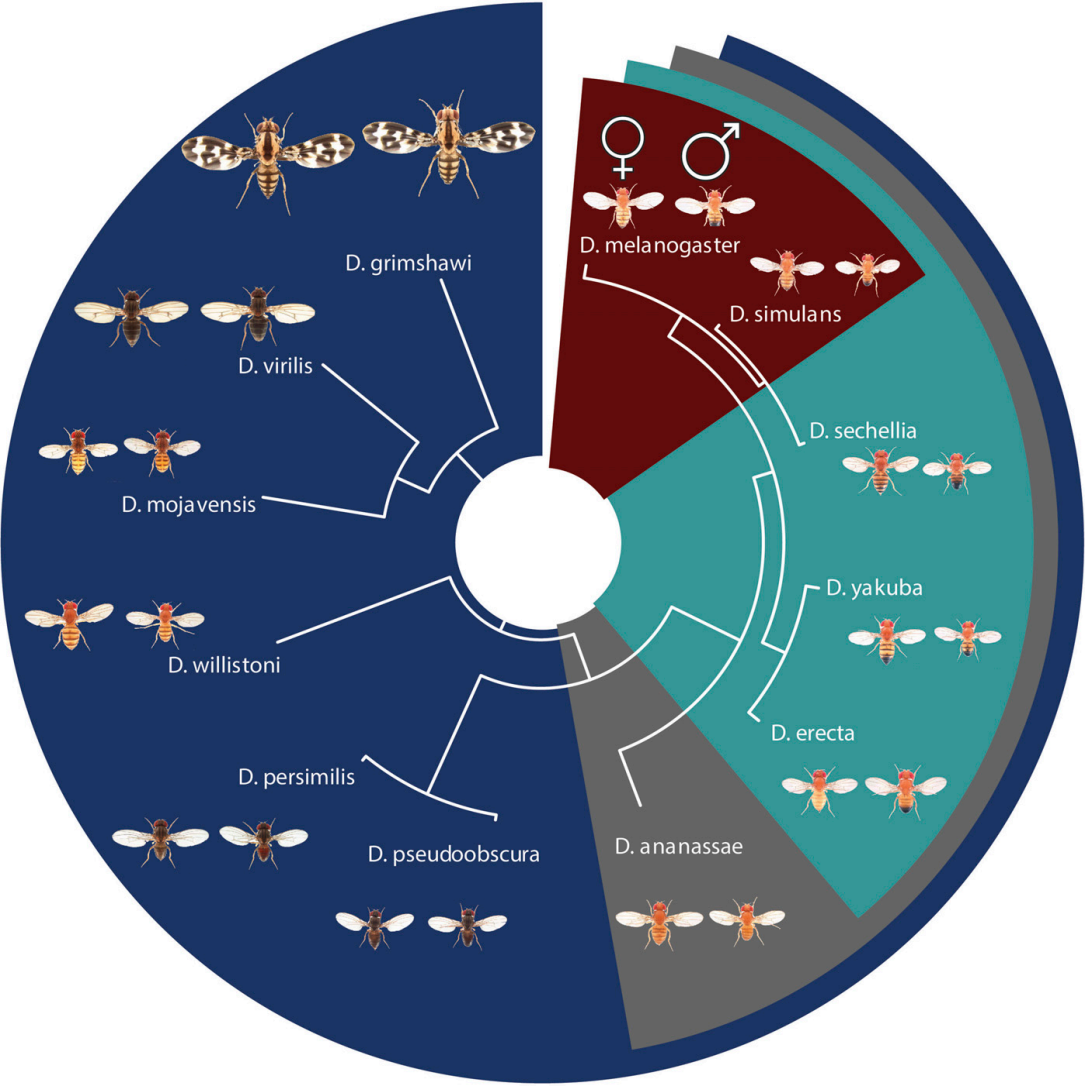
Phylogeny and taxonomic datasets of the 12 Drosophila species used in *flyDIVaS*. Species are organized into four major taxonomic group indicated by color: *D. melanogaster and D. simulans* (*n* = 2, red), melanogaster subgroup (*n* = 5, light blue), melanogaster species group (*n* = 6, gray), 12 *Drosophila* genome species (*n* = 12, dark blue). Males (right) and females (left) of each species are presented and scaled according to their relative size (images generated by Nicolas Gompel).

## Materials and Methods

### Data source

Coding sequences (CDS and their translated protein) of the longest transcript were downloaded from FlyBase R2015_2 (http://www.flybase.org) for each of the 12 sequenced Drosophila species (Clark *et al.* 2007): *D. melanogaster*, *D. simulans*, *D. sechellia*, *D. yakuba*, *D. erecta*, *D. ananassae*, *D. pseudoobscura*, *D. persimilis*, *D. willistoni*, *D. mojavensis*, *D. virilis*, and *D. grimshawi*. These data include the latest genomic release of *D. simulans* (Hu *et al.* 2013) and updated FB2015_02 annotations from NCBI’s GNOMON annotation pipeline (Souvorov *et al.* 2010), which integrates new RNAseq data for nine of the 12 species (*Dmel*, *Dsim*, *Dyak*, *Dere*, *Dana*, *Dpse*, *Dwil*, *Dmoj*, and *Dvir*). The number of unique CDS per species range from 13,920 *in D. melanogaster* to 16,466 in *D. sechellia*.

### Species groupings

Due to divergence and incomplete genome assemblies, greater phylogenetic depth generally results in less alignment coverage. To provide users with a selection of species depths and sequence coverages, we generated four taxonomic datasets (Figure 1): 1) *Dmel-Dsim*; 2) mel subgroup: *Dmel*, *Dsim*, *Dsec*, *Dyak*, and *Dere*; 3) mel group: *Dmel*, *Dsim*, *Dsec*, *Dyak*, *Dere*, and *Dana*; and 4) twelve species: *Dmel*, *Dsim*, *Dsec*, *Dyak*, *Dere*, *Dana*, *Dpse*, *Dper*, *Dwil*, *Dmoj*, *Dvir*, and *Dgri*. The *Dmel-Dsim* species group offers the greatest genomic coverage of 11,278 1:1 orthologs spanning ∼18.6 Mbp, ∼15% of the entire *D. melanogaster* euchromatic genome, while the 12 species set contains 6,040 1:1 orthologs covering ∼11.76 Mbp (Table 1).

**Table 1:** Summary of the four taxonomic datasets used in *flyDIVaS_v1.1*

Taxonomic Dataset	Number of 1:1 Orthologs	Mean Alignment Coverage (%)	Mean *d_N_*/*d_S_* (ω)	Positively Selected Gene Fraction (%)		
				M1a *vs.* M2a (FDR adjusted)	M7 *vs.* M8 (FDR adjusted)	M8 *vs.* M8a (FDR adjusted)
*D. melanogaster* and *D. simulans*	11,278	98.6	0.205	NA	NA	NA
melanogaster subgroup	9,169	95.0	0.129	3.3 (2.4)	4.2 (3.0)	0.2 (0.1)
melanogaster group	8,649	92.1	0.086	2.3 (1.6)	4.4 (3.7)	3.7 (2.2)
*Drosophila* 12 species	6,040	83.9	0.065	0.8 (0.4)	9.6 (5.3)	2.3 (1.0)

Included are orthology and alignment coverages, divergence estimates (*d_N_*/*d_S_*), and fractions of positive selected genes fitted to nested PAML (Yang 1997) models of selection.

### Alignment and analysis

OrthoDB-derived *D. melanogaster* based orthologies for the 12 species were downloaded from FlyBase (Waterhouse *et al.* 2013; dos Santos *et al.* 2015) (gene_orthologs_fb_2015_02.tsv). For each of the four taxonomic groupings, 1:1 *D. melanogaster* pairwise orthologs were collected for divergence and selection analyses. Only genes with a single 1:1 ortholog for each species in that particular dataset were used. For example, the well known developmental gene, *decapentaplegic* (*dpp*), has a single ortholog in all 12 Drosophila species, and subsequently has both alignments and analyses for each of the four taxonomic groupings in *flyDIVaS*. On the other hand, the commonly studied gene, *Alcohol dehydrogenase* (*Adh*), has a duplication in *D. yakuba* and, thus, is not present in any taxonomic datasets other than the *Dmel-Dsim* grouping. A summary of the four 1:1 orthology datasets is found in Table 1.

For each taxonomic dataset, CDS was translated, and sequences were aligned using default parameters in MUSCLE v3.8.31 (Edgar 2004). Amino acid alignments were back-translated to the original CDS sequences and gap-adjusted via perl scripts to retain inframe codons. To reduce alignment errors surrounding insertions and deletions that can negatively affect protein divergence and selection analyses (Markova-Raina and Petrov 2011), we masked +/− three flanking nucleotides at each indel with Ns. Alignment statistics are found in Table 1, and the three generated alignment sets (protein unmasked, CDS unmasked, and CDS masked), as well as unaligned raw fasta files, are available via batch download at *flyDIVaS* (see below).

**Figure 2:**
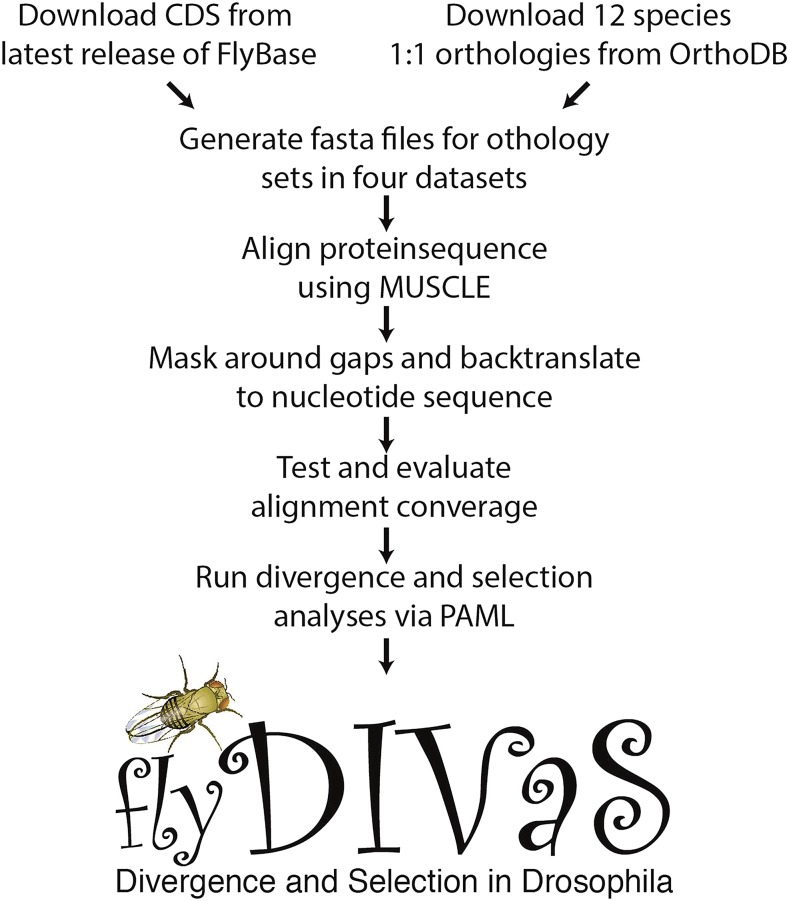
Data flow and analyses in *flyDIVaS*. This database of *Drosophila* divergence and selection is based on 1:1 orthology calls of curated CDS fasta files from species of the 12 *Drosophila* genome project (Clark *et al.* 2007) referenced against *D. melanogaster*.

Estimates of protein divergence and phylogenetic tests of selection are based on a codon substitution framework implemented by PAML (Yang 1997). Rates of CDS/protein evolution (*d_N_*; *d_S_*; *d_N_*/*d_S_*, often referred to as ω) were estimated using PAML model, M0. Tests for selection on protein-coding regions compared three nested pairs of site-specific models: 1) model M1a (neutral) *vs.* M2a (positive selection), 2) model M7 (beta-distributed) *vs.* M8 (beta+ω>1) (Yang 1997), and 3) model M8 (beta+ω>1) *vs.* model M8a (beta+ω=1) (Swanson *et al.* 2003; Wong *et al.* 2004). Confidence values for model comparisons were generated using a likelihood ratio test (LRT) against a χ^2^ distribution. False Discovery Rates (FDR) were generated using the q-value package in R (Storey *et al.* 2015), with significance determined via a corrected *P*-value < 0.01. Figure 2 provides a schematic of the *flyDIVaS* workflow. We stress that divergence estimates and selection tests using the 12 *Drosophila* species dataset should be met with caution due to the saturation of *d_S_* at this phylogenetic distance (see Box 2 in Larracuente *et al.* 2008).

### Database architecture

*flyDIVaS_v1* was developed using an open-source bootstrap architecture, and promotes an interactive user experience through multiple JavaScript plugins. The database is easily updateable and extensible due to an object- (*i.e.*, gene-) centric data structure. The gene-centric schema also decreases computational time required client-side since data files are neither large nor complex. We use a newly available library of open-source JavaScript plugins called BioJS (https://www.biojs.net). These bioinformatics plugins include client-based tools that allow the user to quickly scan the alignment and visualize the percentage conservation at each site. Additionally, we provide an interactive BioJS neighbor-joining tree plugin with collapsible internal nodes. For users with basic informatics skills, *flyDIVaS* provides complete alignment sets (both pre and postmasked alignment files are provided, as are unaligned raw fasta files) and divergence and PAML analysis results for each taxonomic dataset on the Downloads page.

### Data availability

All data necessary for confirming the conclusions presented in the article are represented fully within the article and at the *flyDIVaS* website. The *flyDIVaS* database is freely available for noncommercial use at http://www.flydivas.info.

## Results and Discussion

### flyDIVaS: Divergence and selection in Drosophila

**Figure 3:**
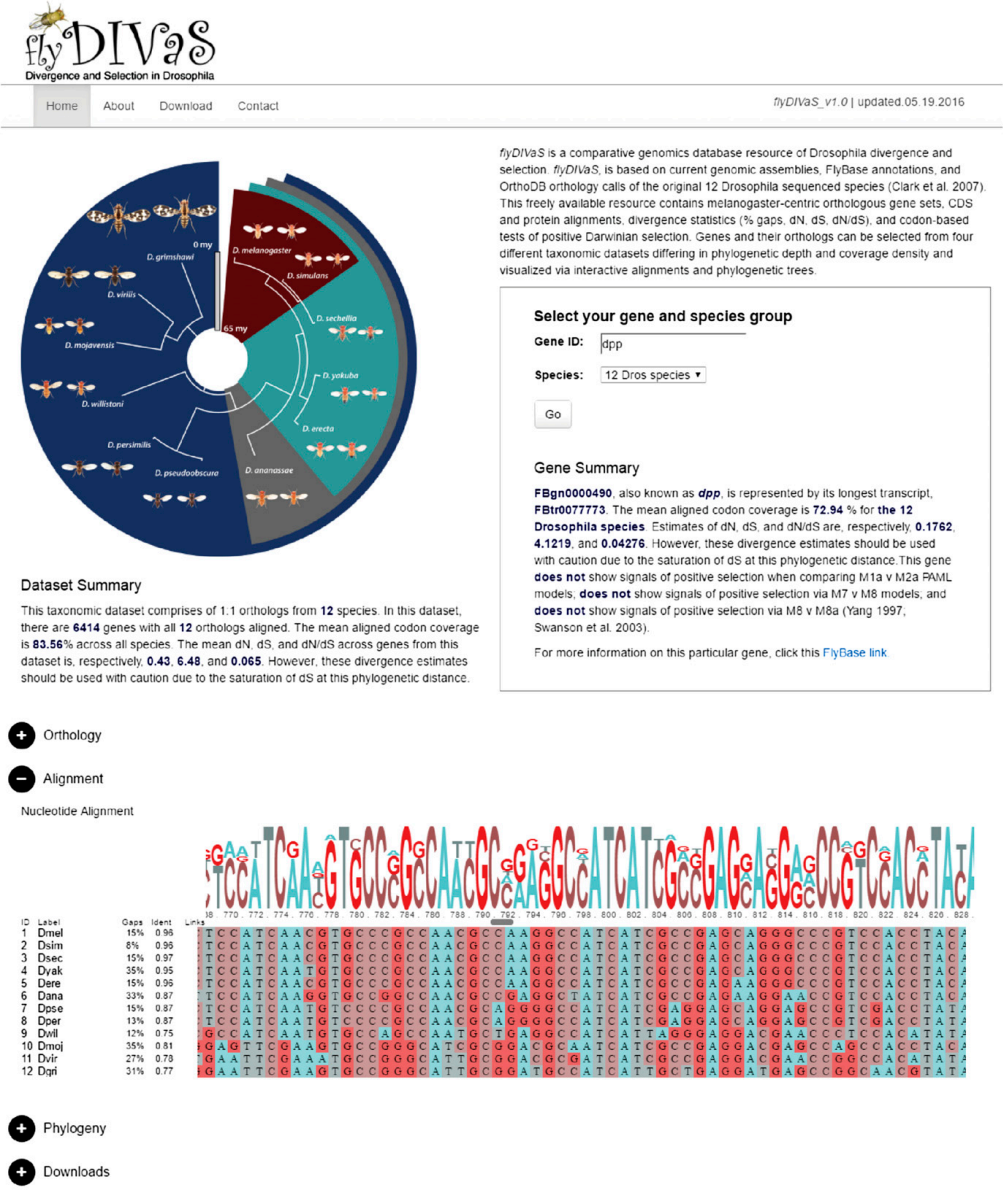
*flyDIVaS* homepage. Search tool allows users to select a Drosophila gene and one of four taxonomic datasets potentially available. Once these parameters are chosen, a summary of the gene and its associated alignment, divergence, orthology, and selection test results are automatically generated. Phylogenetic view changes when a taxonomic dataset is chosen. A summary of orthology, alignment, and divergence is also provided for the chosen dataset. An interactive JavaScript plugin is provided for users to explore alignment characteristics of their selected gene. Features not shown in figure include a gene-specific neighbor-joining tree (Saitou and Nei 1987) of the aligned sequences, and downloadable fasta files. *flyDIVaS* can be accessed at www.flydivas.info.

The genus *Drosophila* provides an ideal model to study the mode and tempo of evolutionary change. Here, we introduce *flyDIVaS*, a new online resource of divergence and selection on protein-coding regions across the fruit fly genus (Figure 3). With a dozen well-assembled and expertly annotated species, relatively small euchromatic genomes, and conserved synteny, *Drosophila* offers a rich trove of data with which to elucidate the molecular and evolutionary mechanisms of conservation and divergence. The initial dataset, generated over a decade ago (Clark *et al.* 2007), was applied to fields as diverse as development, physiology, and cell biology to better understand both pattern and process and, ever since, these data have served as a gold standard for both geneticists and genomicists interested in everything from evolutionary inference to structure–function relationships. Newly assembled species (Hu *et al.* 2013), more comprehensive RNAseq-based annotations (Chen *et al.* 2014), and client-based database platforms, offer a unique opportunity to develop a newly updated comparative genomics resource immediately accessible to a wider cast of researchers and research communities.

*flyDIVaS* is freely available as a user-friendly online interface (www.flydivas.info). As a comparative genomics resource for discovery, *flyDIVaS* generates and provides alignments and selection analyses derived from community-curated resources via user-friendly web tools. The home page is designed to quickly return precomputed data for currently annotated *D. melanogaster* genes using one of four taxonomic datasets of varying phylogenetic depths (2, 5, 6, and 12 species; Figure 3). The user queries a *D. melanogaster* gene, using an auto-fill search tool, based on current FlyBase synonyms from any of three accession types: FlyBase gene symbol, FBgn, or a “CG number”. The “species” dropdown menu automatically populates according to the extent of a gene’s orthology among the 12 *Drosophila* species. Once a gene and its associated dataset are chosen, divergence statistics and links are automatically displayed in the “Gene Summary” section. In addition, basic summary statistics for the entire dataset are shown in the “Dataset Summary” section, found directly below the color-coded, layered phylogeny (Figure 1).

Our original intention was to provide a regularly updated portal for researchers to download comprehensive datasets from this unique comparative genomics resource, with users running analyses via their own inhouse tools. However, most geneticists are interested in a finite set of genes, and/or lack the necessary bioinformatics skills to handle large datasets (Pevzner and Shamir 2009; Welch *et al.* 2014) that are not readily accessible through graphical user interfaces (GUIs). To serve this large segment of the research community, we use the latest offerings of JavaScript tools that are becoming increasingly available to biologists for data integration and visualization. These open-source libraries allow biologists like us, without prior training in web development, to create online portals with the capacity to interactively visualize complex biological data. *flyDIVaS* uses BioJS, an open-source set of JavaScript libraries, to help visualize biological data across alignments and phylogenetic trees (www.biojs.net). *flyDIVaS* applies BioJS in two visual components: 1) an alignment viewer, allowing the user to visualize color-coded alignments of the selected gene, and 2) a basic neighbor-joining phylogeny of the selected gene (Saitou and Nei 1987) allowing users to examine individual characteristics of the gene tree including branch lengths and to compare this gene tree with the canonical species tree (Figure 3). Furthermore, for each gene, we provide raw multi-fasta files for download so that users can perform alignments and analyses using their favorite bioinformatic toolkits.

Integrating such web-friendly tools with large complex datasets may also expedite a much-needed pedagogical shift in the way that big data science such as genomics is taught in the classroom. *flyDIVaS*’ use of client-side processing elicits fast response times and little overhead on the web server, permitting scalable increases in database usage. Users with low broadband width will not suffer from long download times as each precomputed gene file is only ∼4 kB. *flyDIVaS* is particularly compatible with mobile and tablet devices providing accessible platforms in which students and scientists can readily explore comparative and evolutionary analysis results “on the fly”.

In addition to gene-specific queries, *flyDIVaS* provides bulk download access for informatics-savvy users to examine these data, *en masse*. A tarball (tar.gz) for each of the four taxonomic datasets is available on the “Downloads” page. Included are compressed sets of multi-fasta files for each alignment (both masked and unmasked) as well as raw CDS fasta files. *flyDIVaS* also provides tab-separated tables consisting of analysis results for the selection-based models including likelihood values for each of the models, chi-square statistics from the likelihood ratio tests, and both regular and adjusted *P*-values for the model comparisons. The documentation file, “README_flyDIVaS.txt”, found on the Downloads page in flyDIVaS, details the analysis parameters provided.

A major challenge in maintaining an up-to-date and topical genomic database is handling the constant moving targets of updated genome assemblies and annotations. *flyDIVaS* uses an automated pipeline to directly download standardized data from FlyBase for both orthology relationships (originally from OrthoDB) and annotated CDS sequences from the original 12 species. We plan to provide a major release each year, in consultation with FlyBase, with potential new offerings such as evolutionary rate covariation (*e.g.*, Clark *et al.* 2012), network connectivity statistics, and lineage-specific tests of selection, depending on users’ needs.

### Conservation and divergence in Drosophila

Evolutionary rates vary greatly among genes and the proteins they encode. *flyDIVaS*, based on the best assembled and annotated genomes, serves as a foundational data resource for biological discovery. In this next section, we provide a precursory and annotated functional survey of genus-wide divergence and adaptive landscape using *flyDIVaS* data. In each of the four taxonomic datasets, protein divergence is unimodally distributed, but heavily skewed with proteins dispersed along a relatively long tail of high divergence. As the number of species and overall phylogenetic depth increases, both mean *d_N_* and *d_S_* increase (Supplemental Material, Figure S1 and Figure S2) while mean *d_N_*/*d_S_* remains relatively constant (Figure S3), as expected. However, it is clear that the inclusion of more species reduces the overall variance in divergence estimates (Figure S4), highlighting the power of dense phylogenetic coverage such as the data provided by the 12 species dataset (e.g., distribution of P-values for tests of positive selection: Figure S5, Figure S6, Figure S7, and Figure S8).

**Figure 4:**
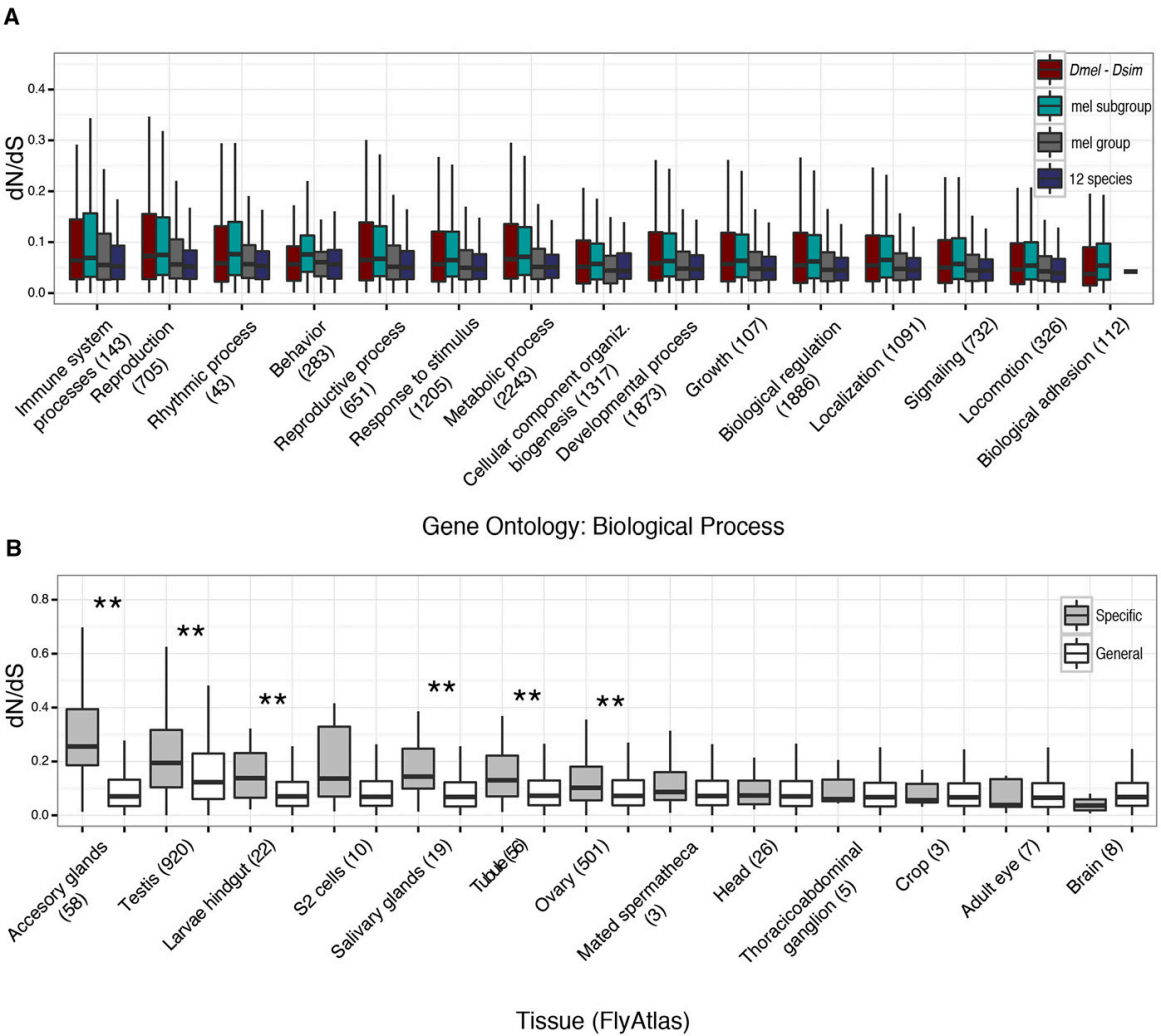
Functional analysis of divergence. (A) Gene ontology comparison for each of the four datasets showing the individual distributions of *d_N_*/*d_S_* in the top hierarchical ontological categories of Biological Process and rank ordered according to divergence. (B) Ranked distribution of *d_N_*/*d_S_* in selected tissues in the melanogaster group dataset. Genes labeled as “general” are expressed in > 50% of the examined tissues (open) and genes labeled as “specific” are expressed in a single tissue (gray). Tissue expression data taken from FlyAtlas (Robinson *et al.* 2013). Astericks denote significant difference between general and specific tissue-expressed genes; ** *P* < 0.001, Wilcoxon rank-sum test.

Our extensive survey of ontologies and tissues also demonstrate that mean rates of amino acid change vary across functional classes (Figure 4A). A large variety of gene ontological (GO) categories are conserved in biological processes (Figure S9), molecular function (Figure S10), and cellular components (Figure S11), as well as FlyBase-defined organismal and developmental ontologies (Figure S12 and Figure S13). Neural tissues including the brain, thoracicoabdominal ganglia, head, and eye contain more conserved genes on average (Figure 4B). In the six species dataset, the most conserved genes with a *d_N_*/*d_S_* of zero (*i.e.*, no replacement changes) are enriched for mitotic and cell cycle processes, as seen in other taxa (Castillo-Davis *et al.* 2004).

Mean evolutionary rate variation among functional ontologies and tissues appears to be driven by the disproportional presence of highly diverged genes in each functional class (Figure 4, Figure S9, Figure S10, Figure S11, Figure S12, Figure S13, Figure S14, Figure S15, Figure S16, and Figure S17). Such rapidly evolving genes and their associated functional classes may play an important role in species-specific differences due to a greater relaxation of selection or adaptation (Singh *et al.* 2012). As reported in previous literature, immune and reproductive ontological classes are among the most rapidly evolving functional groups (Figure 4A and Figure S9). Immune-related genes are hypothesized to coevolve through a continuous arms race with parasitic invaders (Singh and Kulathinal 2000; Wyckoff *et al.* 2002; Schlenke and Begun 2003; Sackton *et al.* 2007; Singh *et al.* 2012). Extracellular proteins—a large component of both immune and reproductive systems—are the most rapidly evolving cellular component class (Figure S13). In addition, the most diverged tissues include such male reproductive tissue as accessory glands and testes (Figure 4, Figure S14, Figure S15, Figure S16, and Figure S17), both involved in sperm development and maturation, and a major component in sperm competition (Clark *et al.* 1995). In fact, the top 10% rapidly evolving proteins are enriched for genes that are upregulated in the testis (*P* < 0.001; χ^2^ ≈ 114.5).

While our results confirm a landscape of functional divergence that highlights the rapid evolution of immune- and reproductive-related traits (Haerty *et al.* 2007; Sackton *et al.* 2007; Singh *et al.* 2012), signals of divergence are strengthened when comparing tissue-specific genes. Figure 4B confirms previous studies in both mammals and *Drosophila* revealing a larger range of mean *d_N_*/*d_S_* estimates among tissue-specific genes compared to genes coexpressed in other tissues (Duret and Mouchiroud 2000; Zhang and Li 2004; Haerty *et al.* 2007; Meisel 2011). For example, the subset of genes solely expressed in a single reproductive tissue (*e.g.*, accessory gland-specific, testis-specific, ovary-specific) has a significantly larger mean *d_N_* and *d_N_*/*d_S_* than genes that are expressed in the same tissue and coexpressed in other tissues (Figure 4B, Figure S14, Figure S15, Figure S16, and Figure S17). On the other end of the distribution, brain-specific genes are less diverged, in agreement with studies in mammals (Duret and Mouchiroud 2000; Wang *et al.* 2007).

The higher tissue-specific divergence pattern can be explained by two alternative hypotheses: 1) less functional pleiotropic constraints, or 2) stronger positive selection. Supporting the latter hypothesis, we found a significant enrichment of positively selected genes in the highest 10% of diverged genes in terms of *d_N_* (M7 *vs.* M8: *P* < 0.001; χ^2^ ≈ 12.7, M7 *vs.* M8a: *P* < 0.001; χ^2^ ≈ 35.0) and *d_N_*/*d_S_* (M7 *vs.* M8: *P* < 0.001; χ^2^ ≈ 48.2, M7 *vs.* M8a: *P* < 0.001; χ^2^ ≈ 96.2) based on the same site-specific phylogenetic selection models (M7vM8 and M7vM8a), and using the six-species melanogaster group dataset. However, whether this adaptive enrichment is driven by biased detection power due to a greater number of substitutions remains to be tested. An enrichment analysis also found a significant overrepresentation of positive selection in testis-specific genes in the M7 *vs.* M8 model test (*P* < 0.01; χ^2^ ≈ 5.5) but not in M7 *vs.* M8a (*P* ≈ 0.25; χ^2^ ≈ 1.32). Interestingly, the most rapidly evolving tissue, accessory glands, was not enriched in genes (either general or tissue-specific) evolving under positive selection (M7 *vs.* M8: *P* ≈ 0.370; χ^2^ ≈ 0.83, M7 *vs.* M8a: *P* ≈ 0.54; χ^2^ ≈ 0.37), indicating a greater role of relaxed selection across this highly divergent class of proteins. Thus, rapidly evolving genes involved in such species-specific traits such as male fertility may be the result of an interplay between neutral and selective forces across a dynamic network of coadapted and newly coopted proteins (Kulathinal and Singh 2012).

### Conclusions

*D. melanogaster* has metamorphosed from a powerful genetic tool into an invaluable genomic model, providing substantive insight across broad biological fields. Much of this transformation was made possible by sequencing related species of the *Drosophila* phylogeny (Clark *et al.* 2007), thereby generating a powerful comparative resource to identify novel functional units in *D. melanogaster* and precipitate new discoveries in evolutionary biology. *flyDIVaS* provides an updated and updatable database of comparative genomics based on the latest assemblies, orthology calls, and expert, community-based annotations of a dozen phylogenetically diverse fruit flies. At www.flyDIVaS.info, users can access gene-specific divergence and selection profiles or download entire comparative genomics datasets from a choice of four taxonomic groups. A preliminary functional survey supports results from previous literature including highly conserved mitotic, cell cycle and neural genes, the rapid evolution of immune and reproductive genes and genetic systems, strong tissue-specific signatures of divergence, and a role for positive selection in driving amino acid divergence in certain tissues. We strongly encourage users to explore their genes, genetic systems, and fly genomes of interest, and to provide comments and requests to improve *flyDIVaS* for its next release.

## Supplementary Material

Supplemental Material
