# Supplementary material for: flyDIVaS: A Comparative Genomics Resource for Drosophila Divergence and Selection
Source: G3 (Bethesda). 2016 May 25;6(8):2355–63. doi: 10.1534/g3.116.031138 (PMC4978890; doi:10.1534/g3.116.031138)
Supplement: Supplemental Material [file supp_6_8_2355__index.html]

flyDIVaS: A Comparative Genomics Resource for Drosophila Divergence and Selection — Supplemental Material 

# *flyDIVaS*: A Comparative Genomics Resource for Drosophila Divergence and Selection

## Supplemental Material for Stanley and Kulathinal, 2016

**Files in this Data Supplement:**

- Figure S1 - Distribution of omega (*d*N) across the four phylogenetic datasets. Color scheme described in Figure 1. (.pdf, 225 KB)
- Figure S10 - Boxplots of divergence estimates based on FlyBase gene ontologies (GO) "Molecular Function" across the four datasets. (.pdf, 292 KB)
- Figure S11 - Boxplots of divergence estimates based on FlyBase gene ontologies (GO) "Cellular Component" across the four datasets. (.pdf, 222 KB)
- Figure S12 - Boxplots of divergence estimates based on FlyBase ontology "Organism" across the four datasets. (.pdf, 245 KB)
- Figure S13 - Boxplots of divergence estimates based on FlyBase ontology "Development" across the four datasets. (.pdf, 263 KB)
- Figure S14 - (.pdf, 350 KB)
- Figure S15 - Distribution of *d*N/*d*S in selected tissues in the melanogaster subgroup dataset. (.pdf, 339 KB)
- Figure S16 - Distribution of *d*N/*d*S in selected tissues in the melanogaster group dataset. (.pdf, 339 KB)
- Figure S17 - Distribution of *d*N/*d*S in selected tissues in the 12 species dataset. (.pdf, 361 KB)
- Figure S2 - Distribution of omega (*d*S) across the four phylogenetic datasets. Color scheme described in Figure 1. (.pdf, 249 KB)
- Figure S3 - Distribution of omega (*d*N/*d*S) across the four phylogenetic datasets. Color scheme described in Figure 1. (.pdf, 248 KB)
- Figure S4 - Violin plot of *d*N/*d*S across the four phylogenetic datasets. Color scheme described in Figure 1. (.pdf, 83 KB)
- Figure S5 - Distribution of p-values from the log-likelihood ratio statistic based on the selection model tests, M1a vs M2a (Yang 1997), for each set of orthologs of a given taxonomic dataset. (.pdf, 187 KB)
- Figure S6 - Distribution of FDR-corrected p-values from the log-likelihood ratio statistic based on the selection model tests, M1a vs M2a (Yang 1997), for each set of orthologs of a given taxonomic dataset. (.pdf, 254 KB)
- Figure S7 - Distribution of p-values from the log-likelihood ratio statistic based on the selection model tests, M7 vs M8 (A-C; Yang 1997) and M8 vs M8a (D-F; Swanson et al. 2003), for each set of orthologs of a given taxonomic dataset. (.pdf, 312 KB)
- Figure S8 - Distribution of FDR-corrected p-values from the log-likelihood ratio statistic based on the selection model tests, M7 vs M8 (A-C; Yang 1997) and M8 vs M8a (D-F; Swanson et al. 2003), for each set of orthologs of a given taxonomic dataset. (.pdf, 349 KB)
- Figure S9 - Boxplots of divergence estimates based on FlyBase gene ontologies (GO) "Biological Process" across the four datasets. (.pdf, 426 KB)
