# Supplementary figures and images for: flyDIVaS: A Comparative Genomics Resource for Drosophila Divergence and Selection
Source: G3 (Bethesda). 2016 May 25;6(8):2355–63. doi: 10.1534/g3.116.031138 (PMC4978890; doi:10.1534/g3.116.031138)

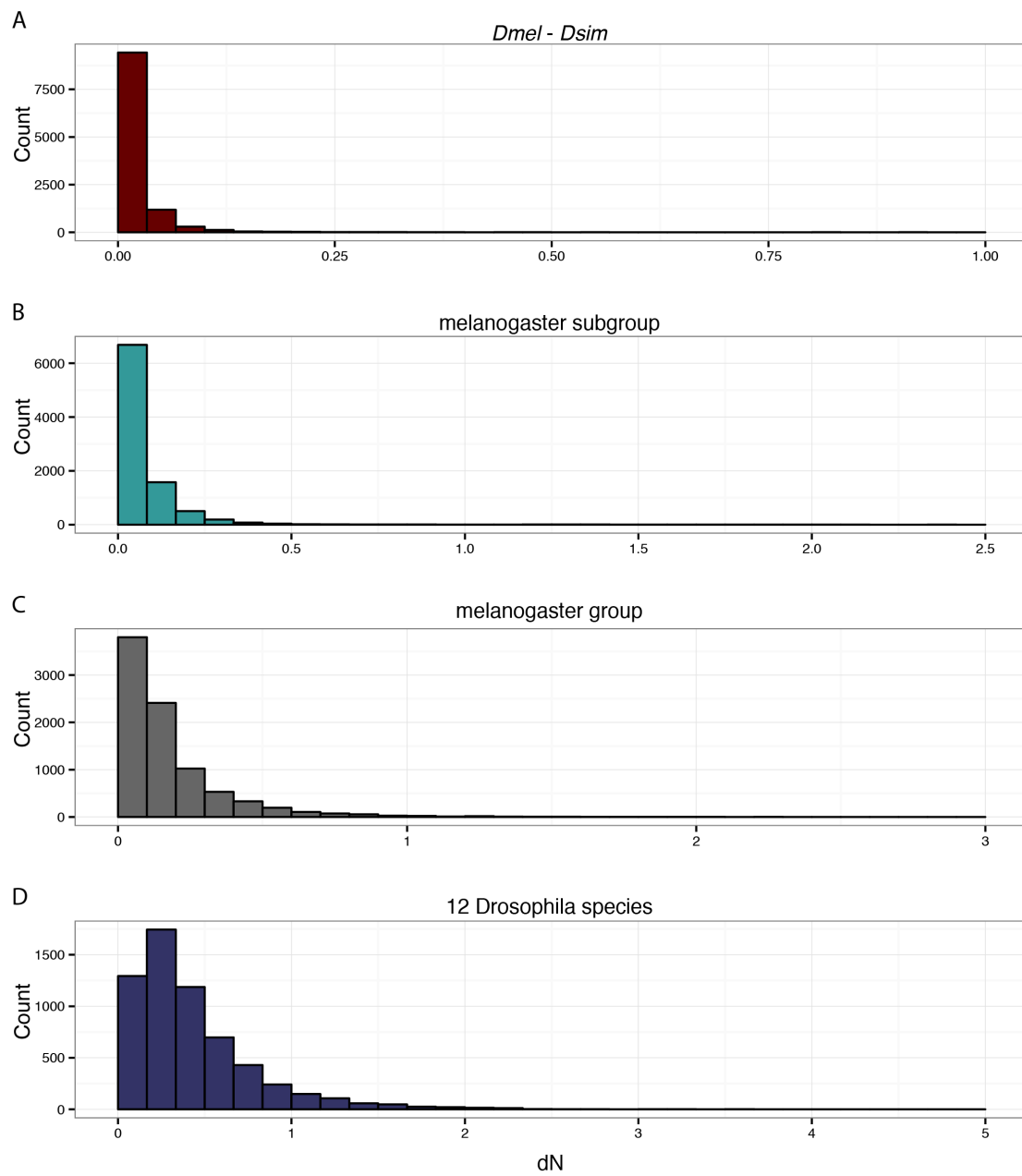

Figure S1

Supplement: Supplemental Material [file supp_g3.116.031138_FigureS1.pdf]

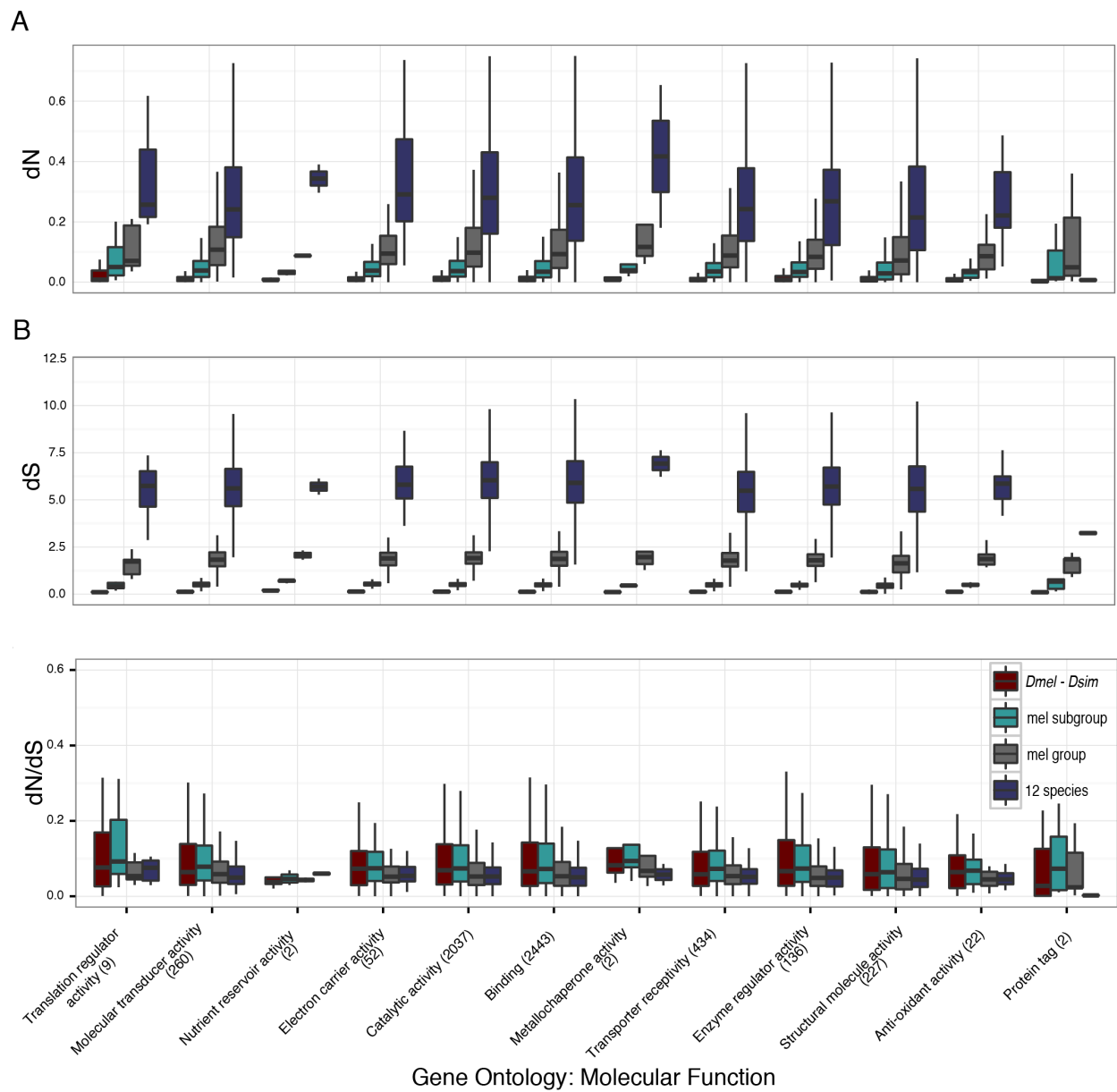

Figure S10

Supplement: Supplemental Material [file supp_g3.116.031138_FigureS10.pdf]

A

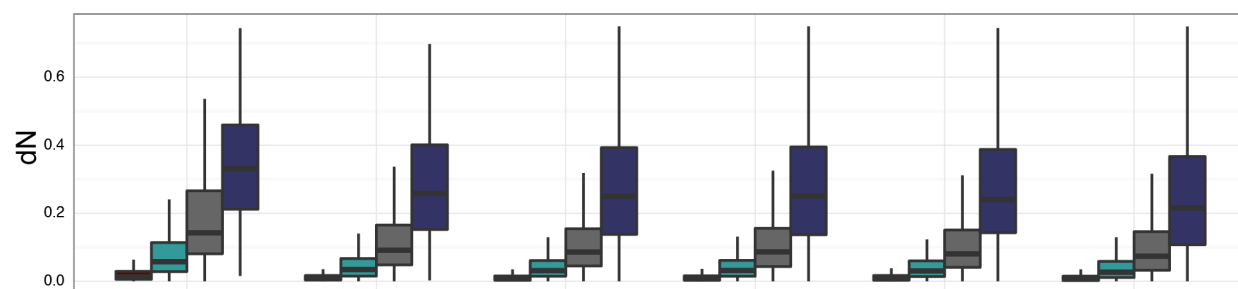

B

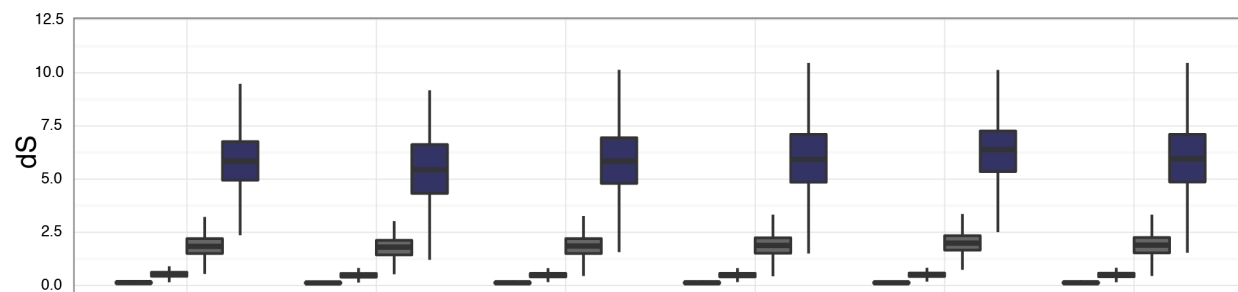

C

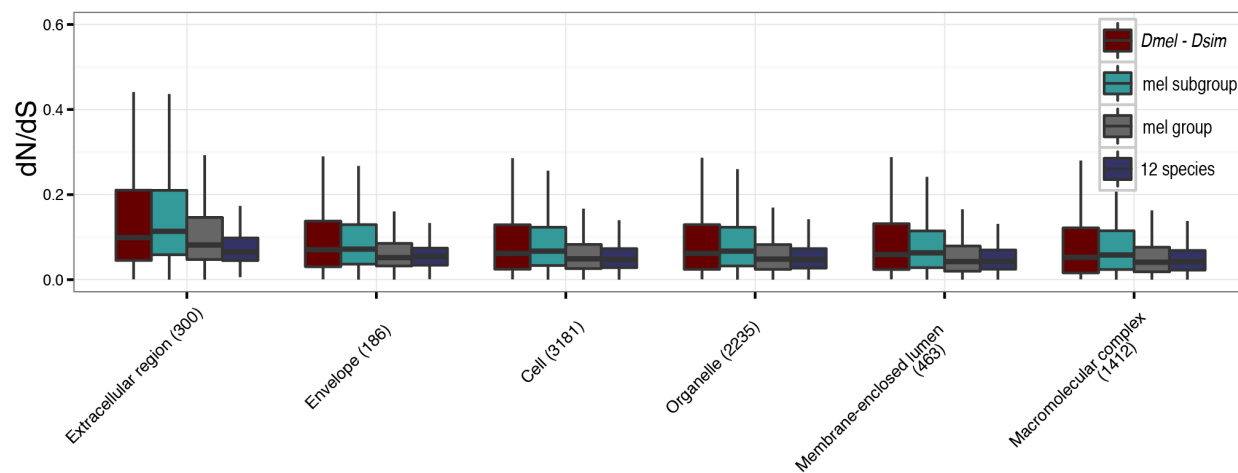

Gene Ontology: Cellular Component

Figure S11

Supplement: Supplemental Material [file supp_g3.116.031138_FigureS11.pdf]

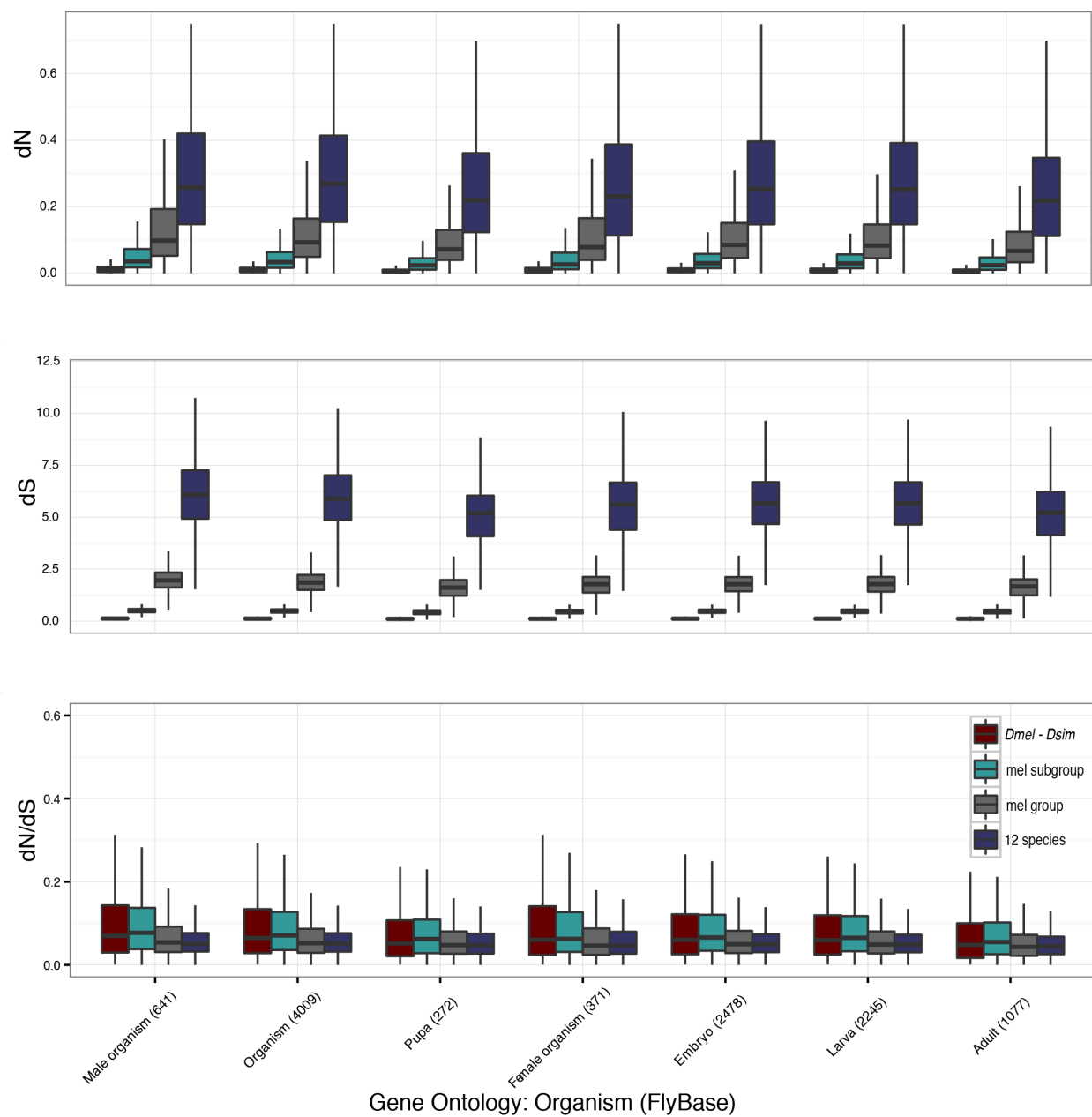

Figure S12

Supplement: Supplemental Material [file supp_g3.116.031138_FigureS12.pdf]

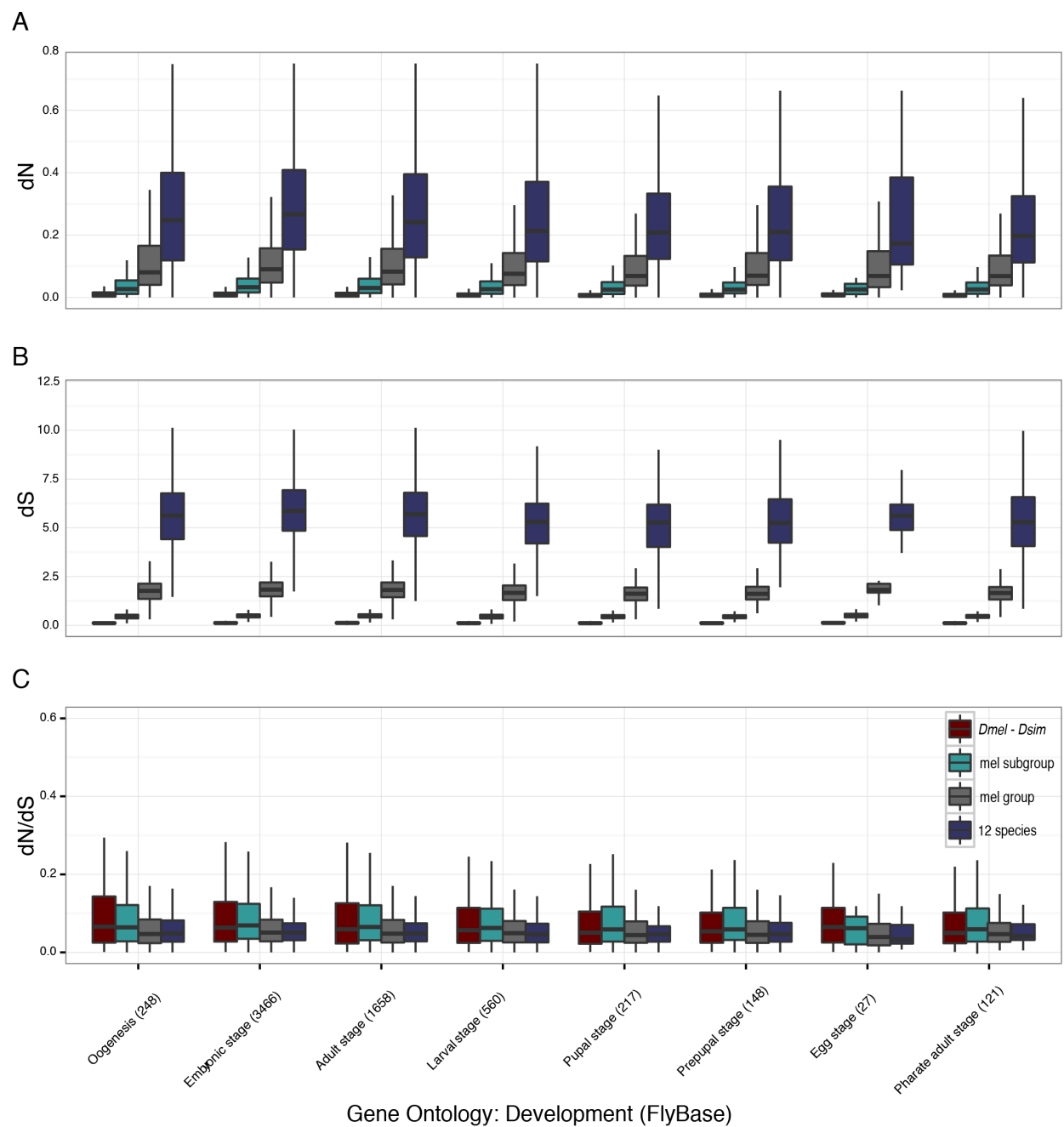

Figure S13

Supplement: Supplemental Material [file supp_g3.116.031138_FigureS13.pdf]

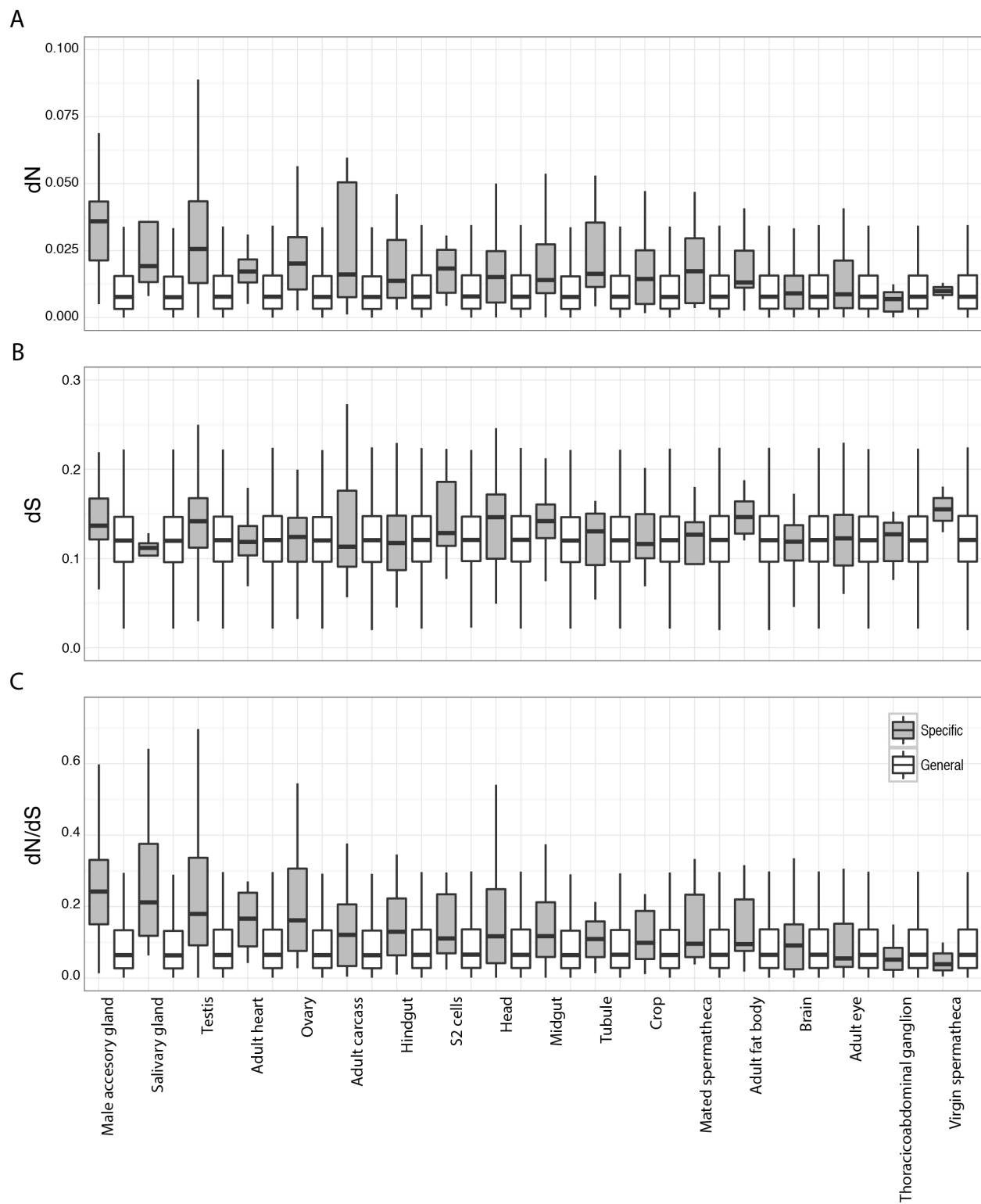

Figure S14

Supplement: Supplemental Material [file supp_g3.116.031138_FigureS14.pdf]

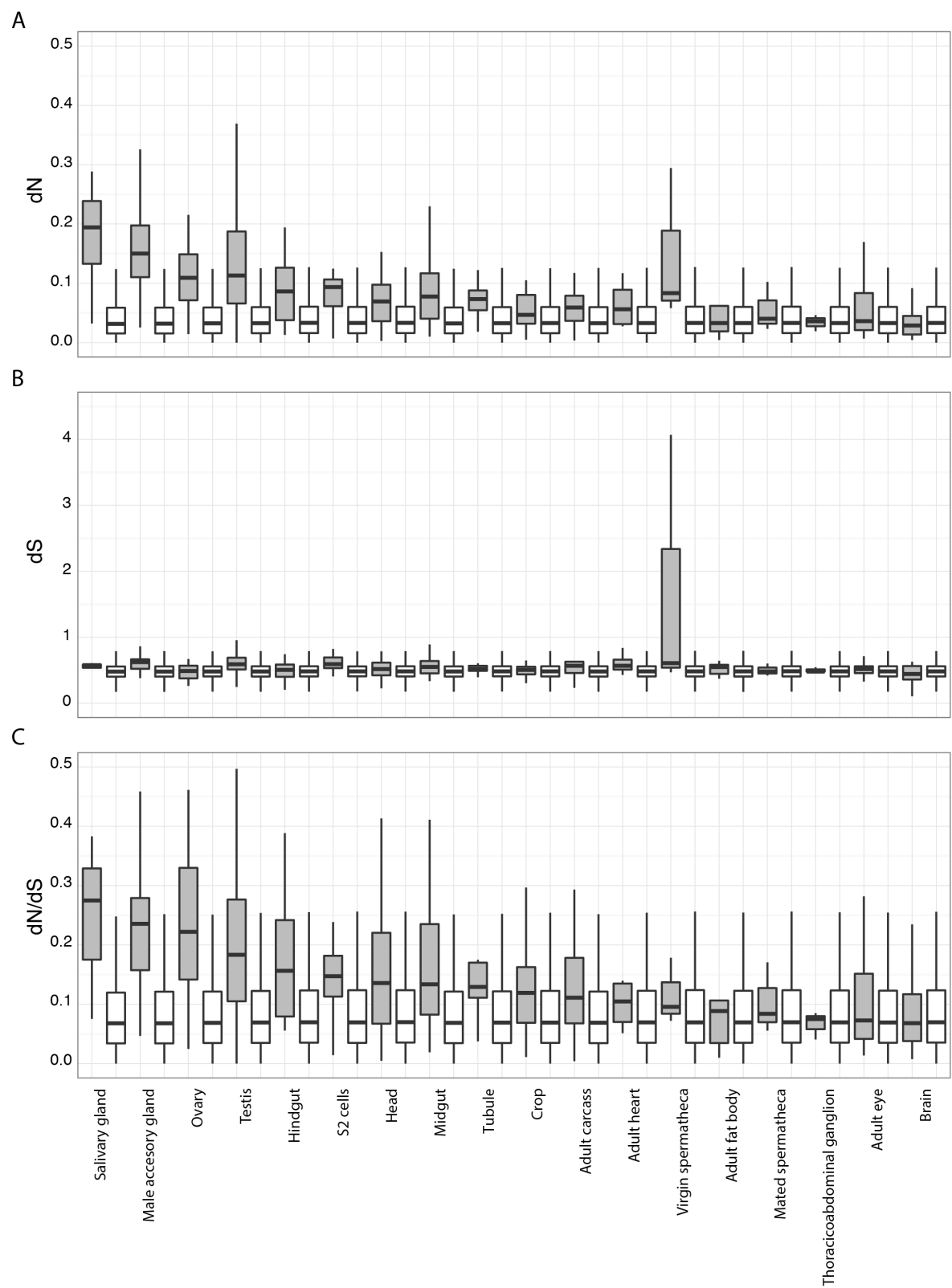

Figure S15

Supplement: Supplemental Material [file supp_g3.116.031138_FigureS15.pdf]

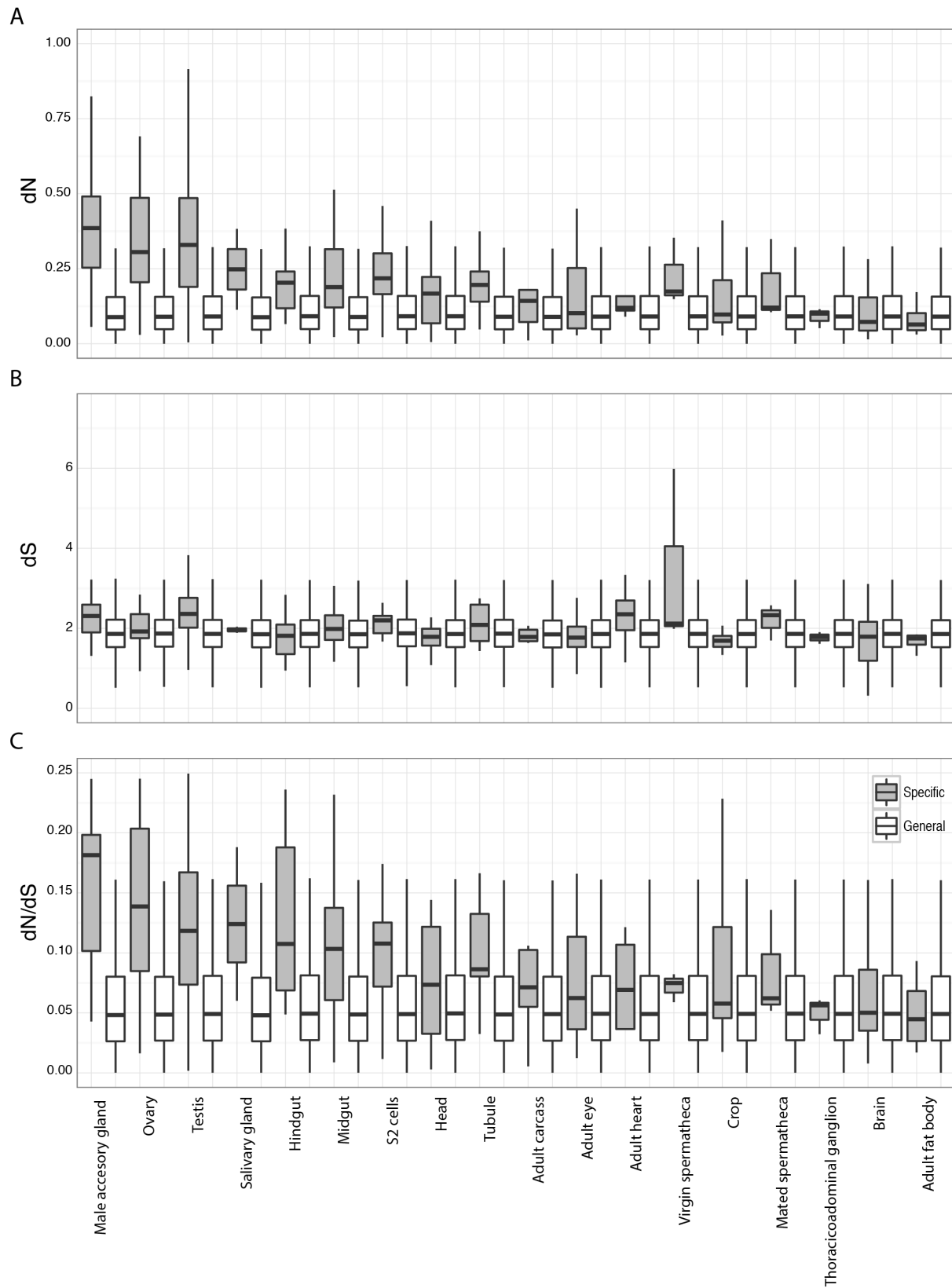

Figure S16

Supplement: Supplemental Material [file supp_g3.116.031138_FigureS16.pdf]

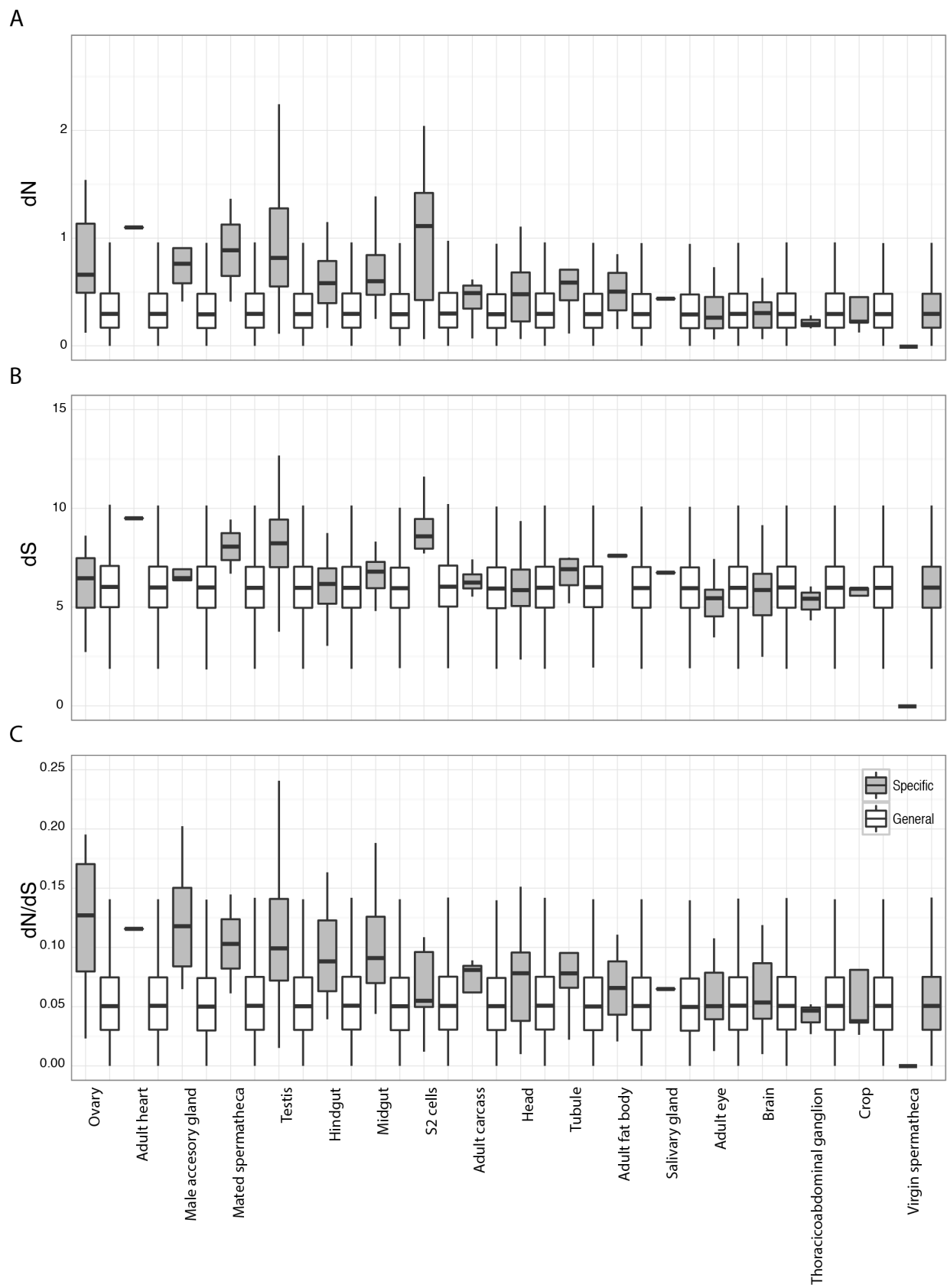

Figure S17

Supplement: Supplemental Material [file supp_g3.116.031138_FigureS17.pdf]

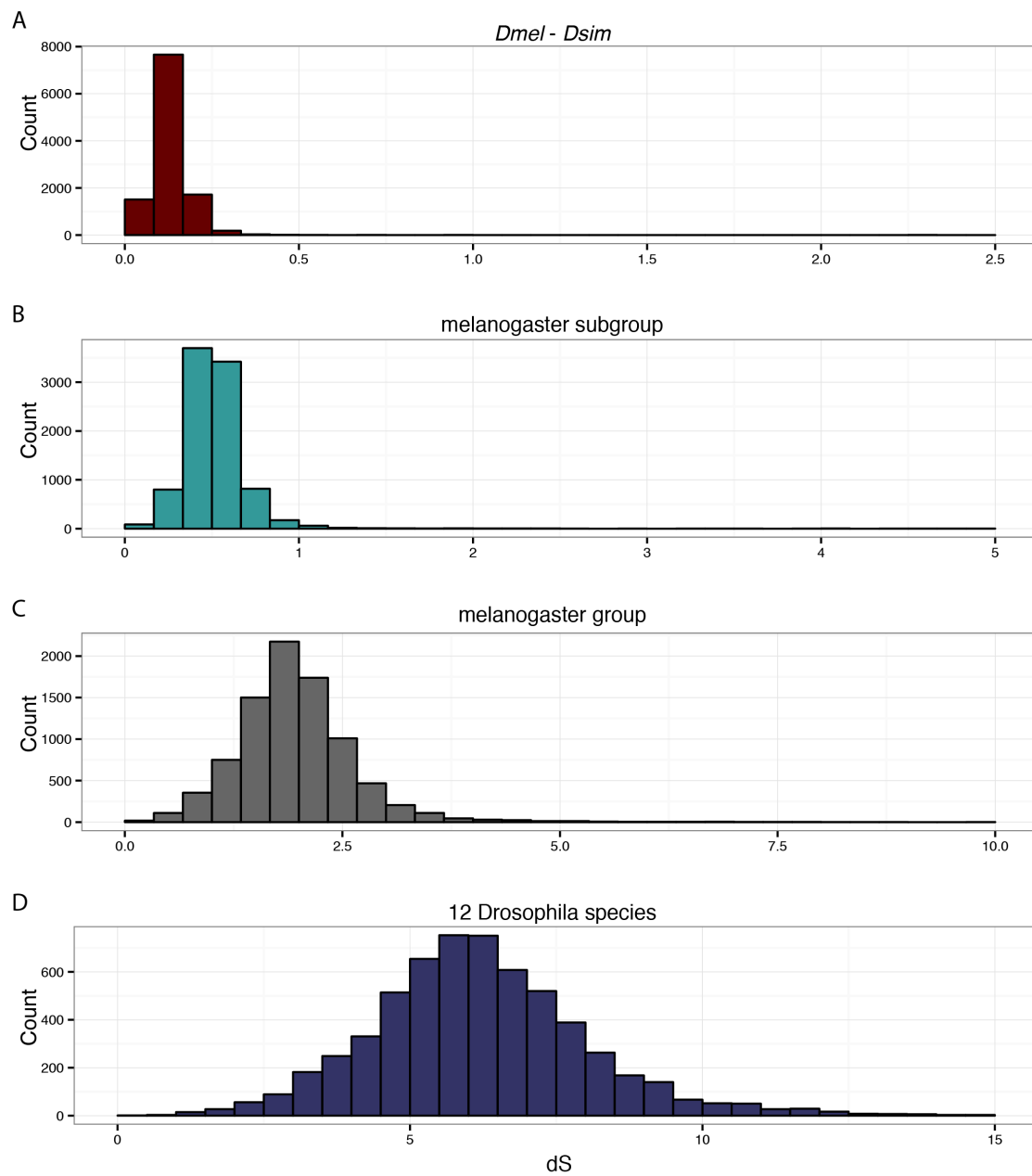

Figure S2

Supplement: Supplemental Material [file supp_g3.116.031138_FigureS2.pdf]

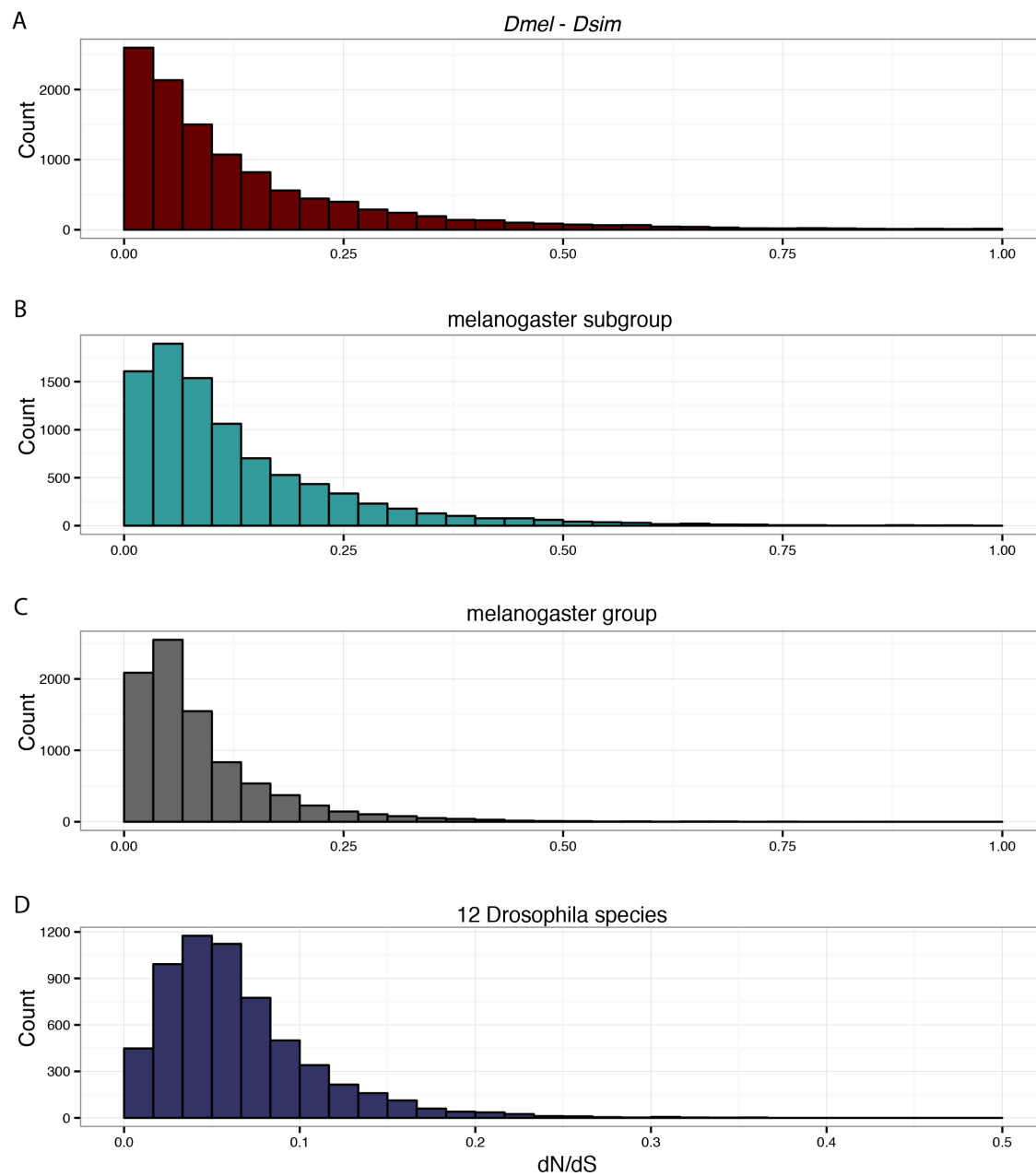

Figure S3

Supplement: Supplemental Material [file supp_g3.116.031138_FigureS3.pdf]

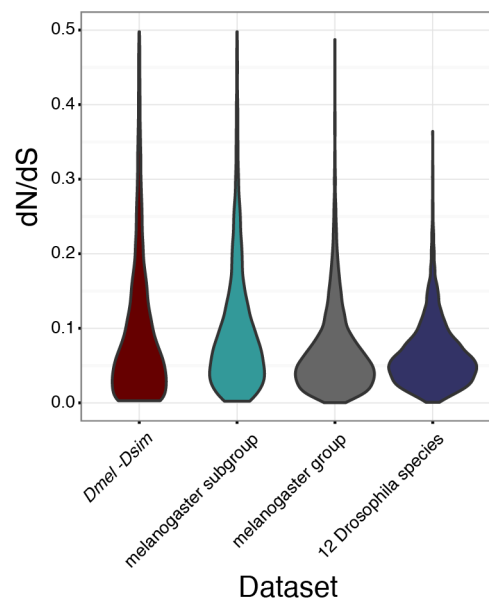

Figure S4

Supplement: Supplemental Material [file supp_g3.116.031138_FigureS4.pdf]

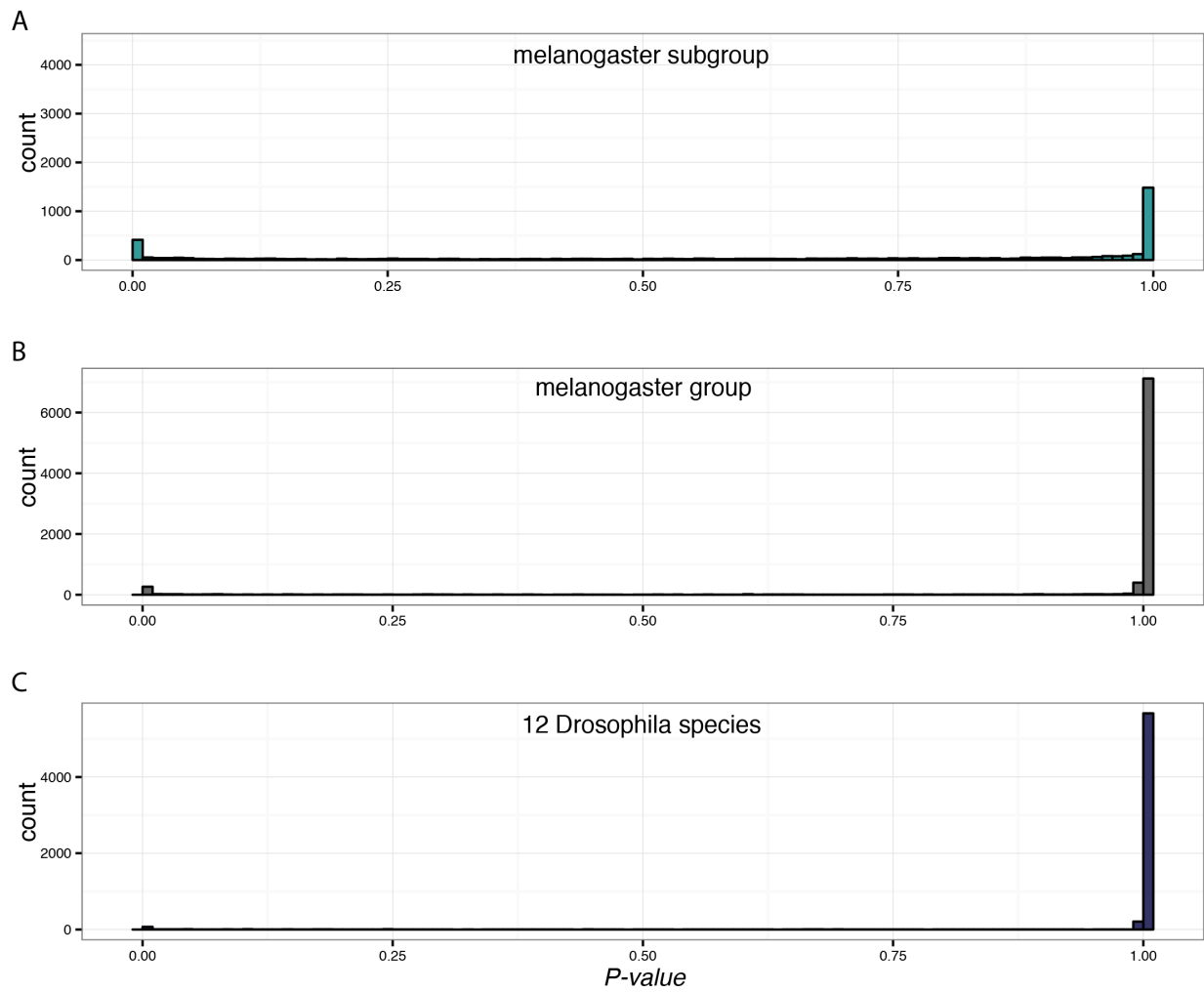

Figure S5

Supplement: Supplemental Material [file supp_g3.116.031138_FigureS5.pdf]

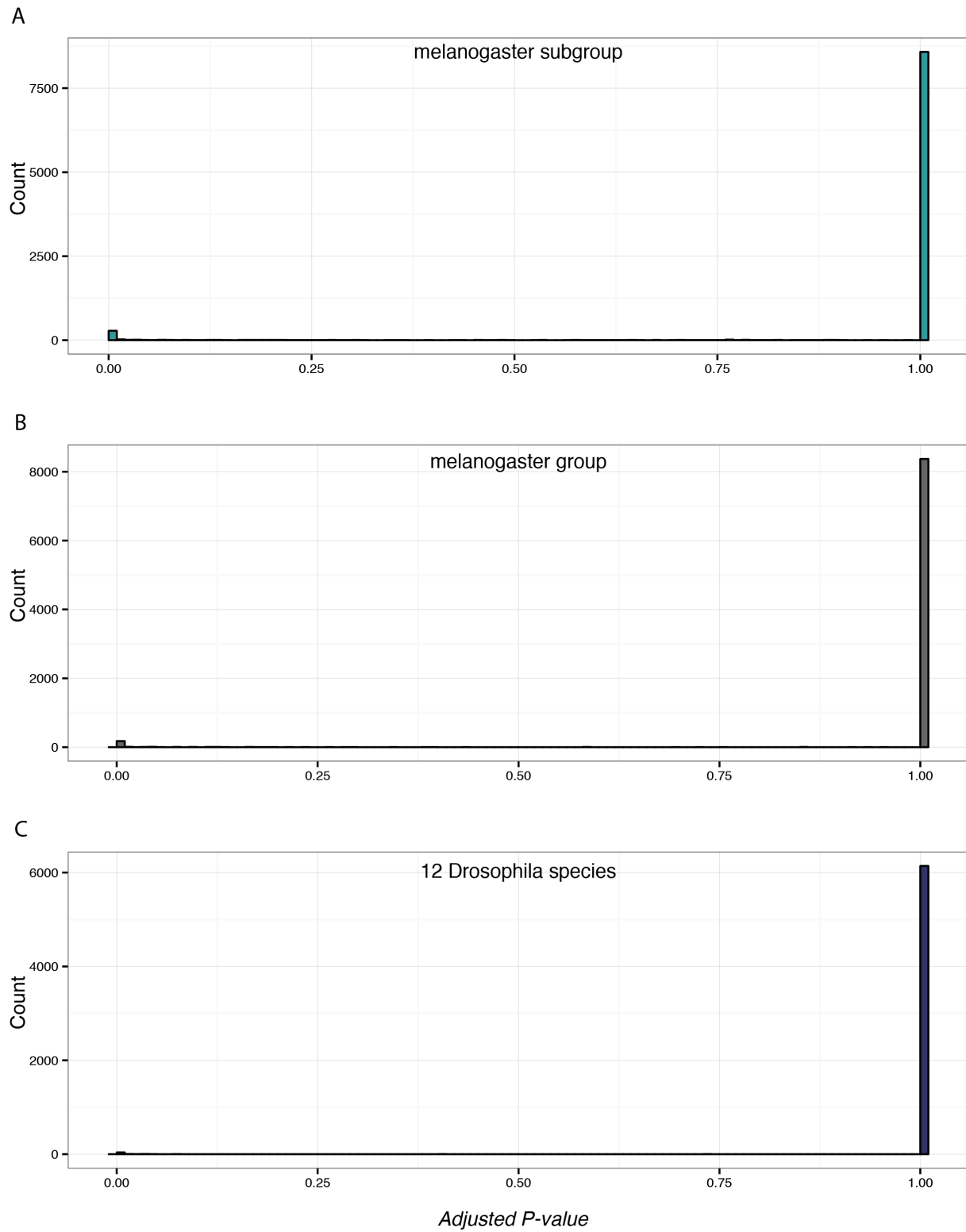

Figure S6

Supplement: Supplemental Material [file supp_g3.116.031138_FigureS6.pdf]

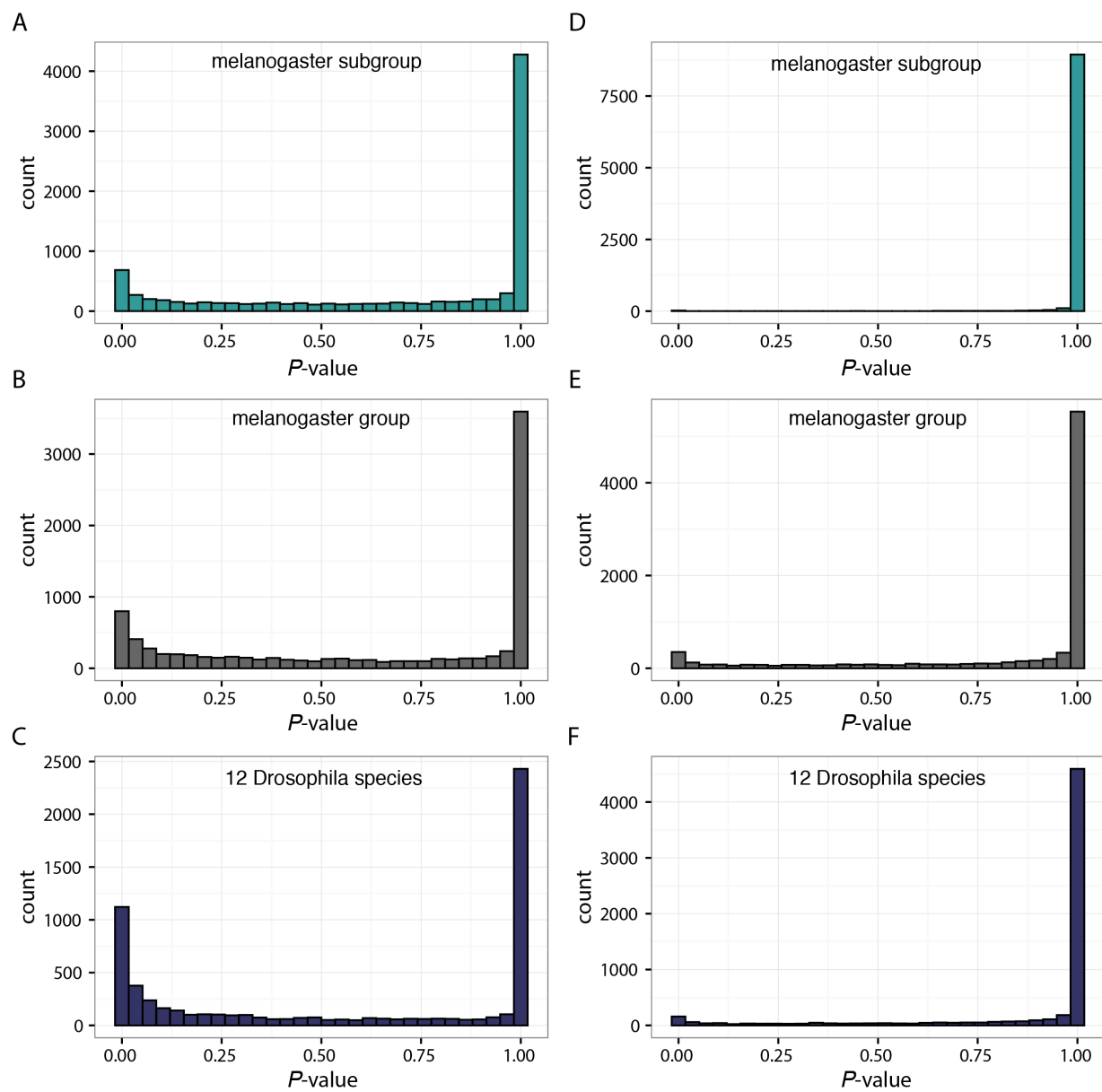

Figure S7

Supplement: Supplemental Material [file supp_g3.116.031138_FigureS7.pdf]

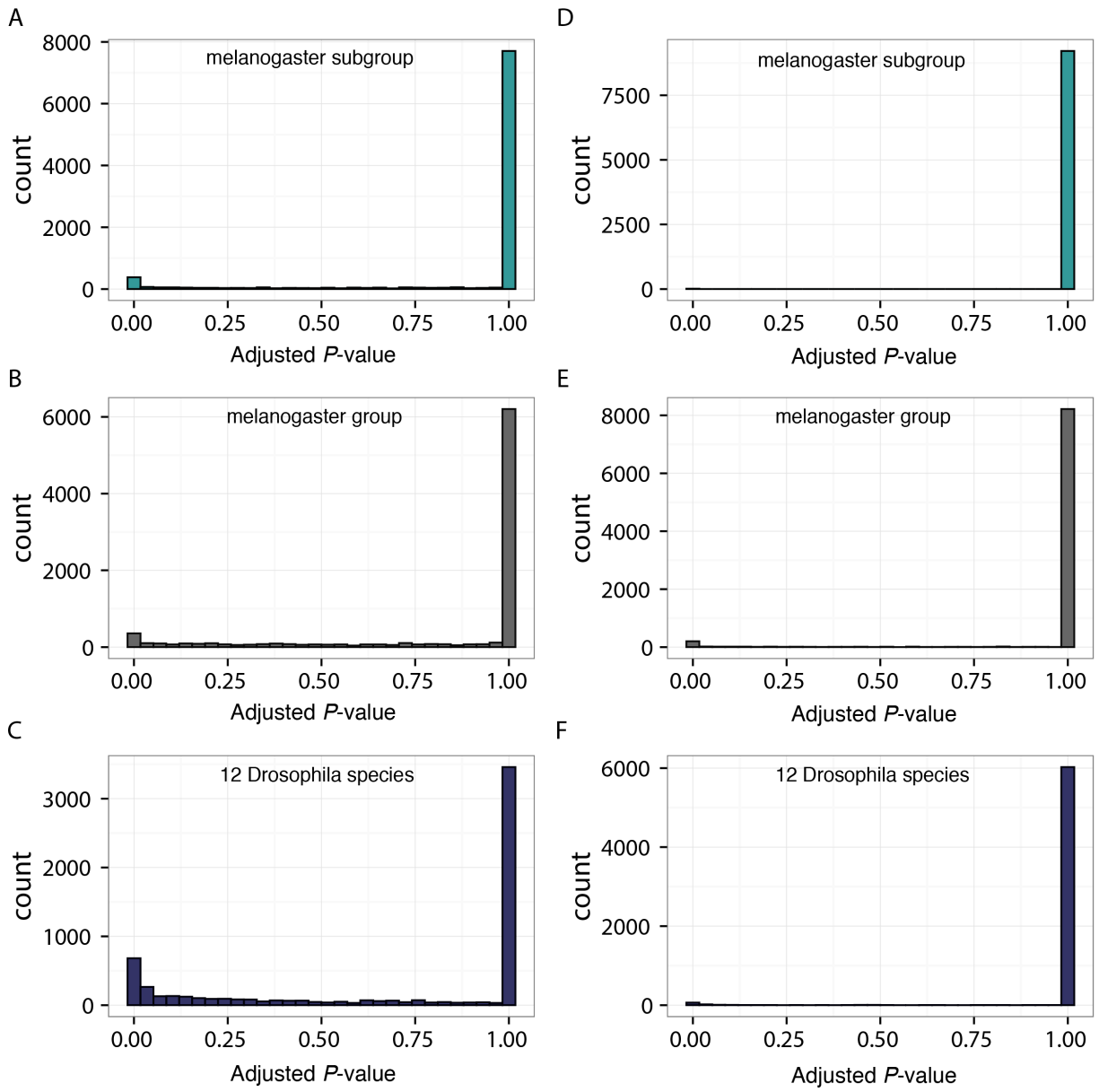

Figure S8

Supplement: Supplemental Material [file supp_g3.116.031138_FigureS8.pdf]

A

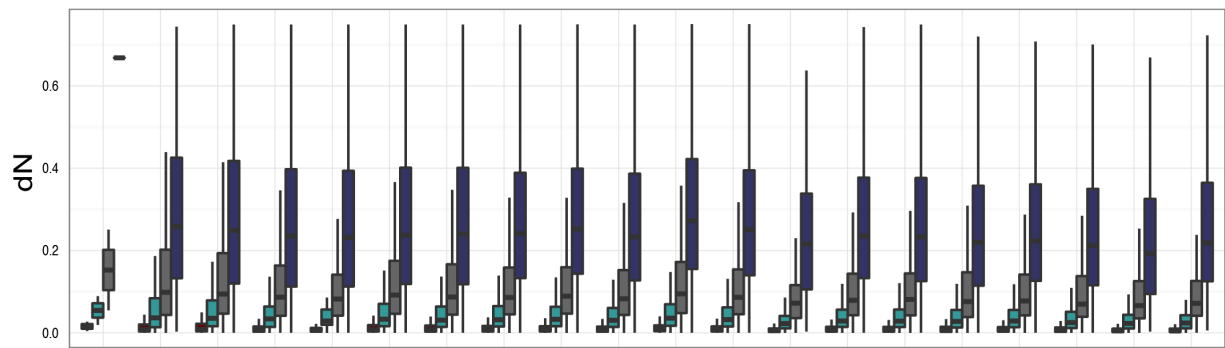

B

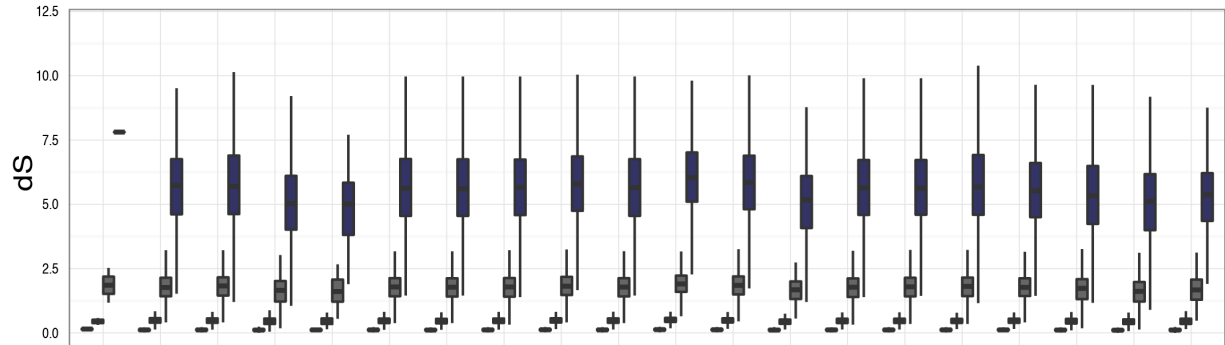

C

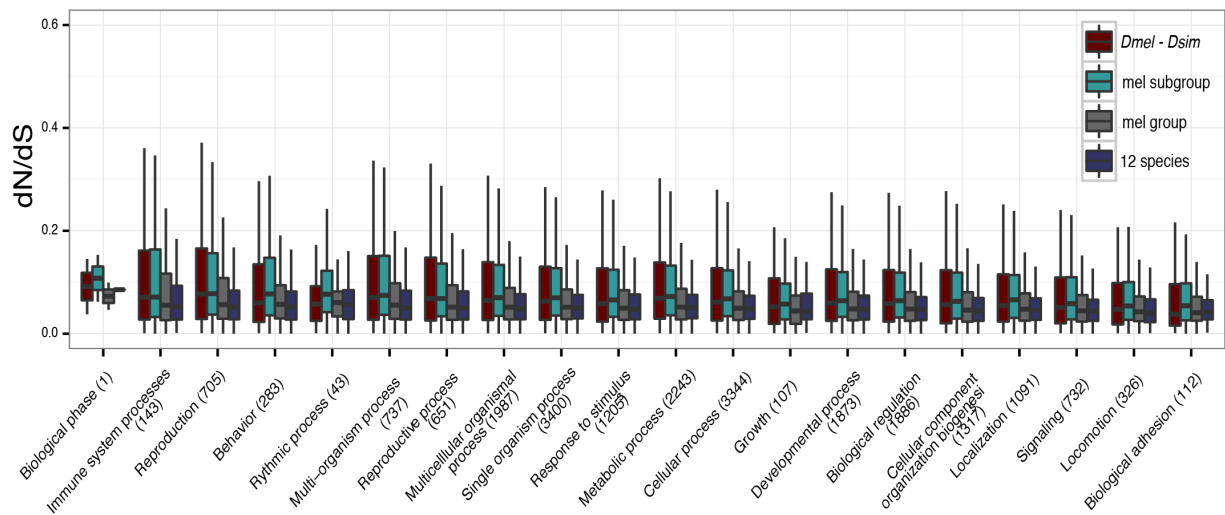

Gene Ontology: Biological Process

Figure S9

Supplement: Supplemental Material [file supp_g3.116.031138_FigureS9.pdf]
